# SOX11-mediated CBLN2 Upregulation Contributes to Neuropathic Pain through NF-κB-Driven Neuroinflammation in Dorsal Root Ganglia of Mice

**DOI:** 10.1007/s12264-025-01530-0

**Published:** 2025-10-29

**Authors:** Ling-Jie Ma, Tian Wang, Ting Xie, Lin-Peng Zhu, Zuo-Hao Yao, Meng-Na Li, Bao-Tong Yuan, Xiao-Bo Wu, Yong-Jing Gao, Yi-Bin Qin

**Affiliations:** Department of Pain Management, Affiliated Hospital of Nantong University, Institute of Pain Medicine and Special Environmental Medicine, Co-innovation Center of Neuroregeneration, Nantong University, Jiangsu, 226001 China

**Keywords:** CBLN2, SOX11, NF-κB, Chemokines, Neuropathic pain

## Abstract

Neuropathic pain, a debilitating condition caused by dysfunction of the somatosensory nervous system, remains difficult to treat due to limited understanding of its molecular mechanisms. Bioinformatics analysis identified cerebellin 2 (CBLN2) as highly enriched in human and murine proprioceptive and nociceptive neurons. We found that CBLN2 expression is persistently upregulated in dorsal root ganglia (DRG) following spinal nerve ligation (SNL) in mice. In addition, transcription factor SOX11 binds to 12 *cis*-regulatory elements within the *Cbln2* promoter to enhance its transcription. SNL also induced SOX11 upregulation, with SOX11 and CBLN2 co-localized in nociceptive neurons. The siRNA-mediated knockdown of *Sox11* or *Cbln2* attenuated SNL-induced mechanical allodynia and thermal hyperalgesia. High-throughput sequencing of DRG following intrathecal injection of CBLN2 revealed widespread gene expression changes, including upregulation of numerous NF-κB downstream targets. Consistently, CBLN2 activated NF-κB signaling, and inhibition with pyrrolidine dithiocarbamate reduced CBLN2-induced pain hypersensitivity, proinflammatory cytokines and chemokines production, and neuronal hyperexcitability. Together, these findings identified the SOX11/CBLN2/NF-κB axis as a critical mediator of neuropathic pain and a promising target for therapeutic intervention.

## Introduction

Neuropathic pain, resulting from damage or dysfunction of the somatosensory nervous system, affects approximately 7%–10% of the global population and remains a major therapeutic challenge due to the inadequate efficacy of current treatments [1]. The dorsal root ganglion (DRG), a critical relay center for nociceptive signal transmission, exhibits extensive molecular and cellular remodeling following peripheral nerve injury, driving maladaptive plasticity within pain pathways [2]. Although transcriptional reprogramming in DRG neurons is known to contribute to neuropathic pain pathogenesis [3], the key molecular regulators and their downstream effectors remain incompletely understood. Emerging spatial transcriptomics and cross-species DRG atlas studies have identified novel molecular candidates involved in sensory processing [4–6]; however, their precise functional roles in the development and maintenance of neuropathic pain have yet to be systematically elucidated.

The cerebellin (CBLN) family, consisting of CBLN1–4, plays critical roles in synapse formation and plasticity within the central nervous system [7]. While CBLN1 and CBLN4 have been implicated in cerebellar function and neuropsychiatric disorders, respectively [8, 9], the role of CBLN2—a protein predominantly enriched in the forebrain—in nociceptive processing remains poorly understood. Intrathecal administration of synthetic CBLN1 or CBLN2 induces mechanical hypersensitivity [10], and mass spectrometry has detected CBLN1, CBLN2, and CBLN4 peptides in the mouse spinal cord [11]. However, the expression profile, functional contribution, and regulatory mechanisms of CBLN2 in the DRG following peripheral nerve injury have not been investigated.

Transcriptional reprogramming of injury-responsive genes in DRG neurons constitutes a fundamental mechanism driving neuropathic pain pathogenesis [12]. Among injury-induced transcription factors, SRY-box transcription factor 11 (SOX11) has emerged as a key regulator with a dual role in promoting axon regeneration and maintaining chronic pain through chromatin remodeling [13, 14]. Bioinformatic analyses have revealed that SOX11 binding motifs are significantly enriched in the promoters of genes involved in synaptic transmission and neuronal excitability [15, 16]. However, whether SOX11 regulates cerebellin family members, including CBLN2, remains unknown. Furthermore, the specific downstream effectors through which SOX11 mediates persistent maladaptive changes in pain circuits are still unclear.

While the nuclear factor kappa B (NF-κB) pathway is established as a master regulator of neuroinflammatory responses in neuropathic pain—driving cytokine production and neuronal hyperexcitability [17], the upstream signals triggering neuronal NF-κB activation are not fully characterized, particularly beyond the canonical TNF-α/IL-1β-mediated mechanisms [18]. To address these gaps, we employed an integrative approach combining spatial transcriptomics, *cis*-regulatory analysis, electrophysiological recording, and behavioral assessments. Our findings reveal the SOX11-CBLN2 axis as a previously unrecognized upstream activator of NF-κB signaling in the DRG, identifying this cascade as a promising therapeutic target for neuropathic pain management.

## Materials and Methods

### Animals and Surgery

Adult male ICR mice (6–8 weeks, 25 ± 5 g) were obtained from the Experimental Animal Center of Nantong University (Jiangsu, China). Mice were housed under standard conditions (22 ± 1 °C, 55% ± 5% humidity, 12 h light/dark cycle) with free access to food and water. All procedures were approved by the Animal Ethics Committee of Nantong University and complied with the International Association for the Study of Pain (IASP) guidelines.

The L4 spinal nerve ligation (SNL) was performed as previously described [19]. Briefly, following dorsal fur removal with an electric clipper, mice were anesthetized in a chamber with 2% isoflurane (4 L/min fresh gas flow, 0.41 mL/min anesthetic rate) until loss of righting reflex, then maintained via facemask (1 L/min gas flow). Body temperature was stabilized at 37 °C using a heating pad. After dorsal disinfection with iodine and ethanol, a midline incision was made at the iliac crest. The L5 transverse process was removed to expose L3-L4 spinal nerves, and the L4 nerve was tightly ligated with 6-0 silk suture (sham group: exposure without ligation). Incisions were sutured and disinfected, with postoperative monitoring for motor function and wound healing.

### Drugs and Administration

The siRNA targeting *Cbln2* and *Sox11* (Table 1) and additional scrambled siRNA were synthesized by GENCEFE Biotech (Wuxi, China). Recombinant CBLN2 protein (7044-CB-050), NF-κB inhibitor PDTC (S3633), and MEK inhibitor PD98059 (513000) were purchased from R&D Systems (Minneapolis, MN, USA), Selleck Chemicals (Houston, Texas, USA), and Merck Millipore (Darmstadt, Germany), respectively. PDTC and PD98059 were dissolved in 1% DMSO (Sigma, D5879, St. Louis, MO, USA).

**Table 1:** The sequences of siRNAs

Target		Sequences
*Cbln2*	siRNA	5′-GCA ACC GTA CCA TGA CCA T-3′
*Sox11*	siRNA-1	5′-GCT TCA AGA ACA TCA CCA A-3′
	siRNA-2	5′-CGA TGA AGA CGA CGA CGA A-3′
	siRNA-3	5′-GCT GGA AGA TGC TGA AGG A-3′
*Scramble*	siRNA	5′-TTC TCC GAA CGT GTC ACG T-3′

Intrathecal injection was performed as described previously [20]. In brief, mice were anesthetized with 2% isoflurane (RWD Life Science, R510-22, China) and placed in a constant temperature cushion. A 30-G needle (Hamilton, Reno, USA) was inserted at a 35°–45° angle into the cerebrospinal fluid between the L4–L5 intervertebral space. Successful injection was confirmed by tail flicking.

### Nociceptive Behavioral Assessment

Mechanical sensitivity was assessed using von Frey filaments (Stoelting, USA) applied to the plantar surface of the hind paw. Mice were acclimated to testing chambers (20 cm × 15 cm × 15 cm) for 1 h/day over three days. Paw withdrawal threshold (PWT) was measured using logarithmically incremental stiffness of von Frey filaments (0.02 g, 0.04 g, 0.07 g, 0.16 g, 0.40 g, 0.60 g, 1.0 g, and 2.0 g) according to Dixon’s up-and-down method [21].

A radiant heat source (IITC model 390 Analgesia Meter, Life Science, USA) was focused on the hind paw, and withdrawal latency (PWL) was measured. Mice were put in a plastic box placed on a glass plate and allowed 30 min to acclimate. A 20-s cutoff prevented tissue damage. Latencies were averaged in three tests with a 5-min interval. All tests were performed blindly.

### RNA Isolation and qRT-PCR

Total RNA was isolated from DRG using TRIzol reagent (Invitrogen/Thermo Fisher Scientific, 15596018, USA). Reverse transcription was performed with PrimeScript™ RT Kit (Takara, RR047A, Japan). qRT-PCR utilized TB Green™ Premix (Takara, RR820A, Japan) on a StepOnePlus system (Applied Biosystems, USA). Primer sequences are listed in Table 2. PCR amplification was carried out at 95 °C for 30 s, followed by 40 cycles of cycling at 95 °C for 10 s and 60 °C for 30 s. Data were normalized to *Gapdh* and analyzed using the 2^−ΔΔCt^ method.

**Table 2:** The sequences of primers

Target		Sequences
*Cbln2*	Forward	5′-CAT TTC TGT GCG CTC AGG CAG T-3′
	Reverse	5′-ACT GGA GGC AAG GTC AAA GTG G-3′
*Sox11*	Forward	5′-GGA CCT GGA TTC CTT CAG CGA G-3′
	Reverse	5′-GTG AAC ACC AGG TCG GAG AAG T-3′
*Cxcl2*	Forward	5′-CAT CCA GAG CTT GAG TGT GAC G-3′
	Reverse	5′-GGC TTC AGG GTC AAG GCA AAC T-3′
*Cxcl3*	Forward	5′-TGA GAC CAT CCA GAG CTT GAC G-3′
	Reverse	5′-CCT TGG GGG TTG AGG CAA ACT T-3′
*Cxcl9*	Forward	5′-CCT AGT GAT AAG GAA TGC ACG ATG-3′
	Reverse	5′-CTA GGC AGG TTT GAT CTC CGT TC-3′
*Ccl3*	Forward	5′-ACT GCC TGC TGC TTC TCC TAC A-3′
	Reverse	5′-ATG ACA CCT GGC TGG GAG CAA-3′
*Ccl4*	Forward	5′-ACC CTC CCA CTT CCT GCT GTT T-3′
	Reverse	5′-CTG TCT GCC TCT TTT GGT CAG G-3′
*Gapdh*	Forward	5′-AAA TGG TGA AGG TCG GTG TGA AC-3′
	Reverse	5′-CAA CAA TCT CCA CTT TGC CAC TG-3′

### Western Blot

DRG tissues were homogenized in RIPA buffer (Solarbio, R0010, China) using a tissue grinder. For cell samples, lysates were incubated on ice for 30 min, centrifuged at 15,000 r/min for 20 min, and supernatants were collected. Protein concentration was determined via the BCA assay (Pierce™, USA). Protein samples (30 or 60 μg/lane) were mixed with loading buffer, denatured at 95°C, and separated on a 10% SDS-PAGE gel. Proteins were transferred to a PVDF membrane (Millipore, USA) using a wet transfer system. Membranes were blocked with 5% skim milk (in TBST) and incubated overnight at 4°C with the following primary antibodies: CBLN2 (Rabbit, 1:800; Invitrogen, PA5-101514, USA), GAPDH (Mouse, 1:20,000; Millipore, MAB374, USA), NF-κB p65 (Rabbit, 1:1000; CST, 8242S, USA), Phospho-NF-κB p65 (Rabbit, 1:1000; CST, 3033S, USA). After washing, membranes were incubated with IRDye 800CW-conjugated secondary antibodies (1:10,000; Li-COR Bioscience, 926-32213/32210, USA) for 2 h at room temperature. Signals were quantified using ImageJ (NIH, Bethesda, MD, USA) with Odyssey CLx Imaging System (Li-COR Bioscience, USA).

### Enzyme-linked Immunosorbent Assay (ELISA)

Mouse CBLN2 ELISA kit was purchased from LunChangShuo Biotech (WED-24058, China). Animals were transcardially perfused with PBS at 10 d after SNL. DRG tissues were homogenized in RIPA buffer (Solarbio, R0010, China) containing phosphatase and protease inhibitors (Sigma-Aldrich, MS-SAFE, USA). Protein concentrations were determined using the Pierce™ BCA Protein Assay Kit (Thermo Fisher Scientific, 23225, USA). For each reaction in a 96-well plate, 100 μg of protein was used, and ELISA was performed according to the manufacturer’s protocol. A standard curve relating the OD values to the concentration of CBLN2 (pg/mL) in the standard solutions was plotted, and an equation of the curve was obtained.

### Immunohistochemistry

After cardiac perfusion with 0.01 mol/L PBS and 4% paraformaldehyde (PFA), L4 DRG were dissected from mice, post-fixed for 6 h, dehydrated in 30% sucrose, and sectioned into 14 μm frozen slices. Sections were blocked with 1% BSA and incubated with the following primary antibodies: CBLN2 (Rabbit, 1:500; Invitrogen, PA5-101514, USA), neuronal class III β-tubulin (TUJ1; Mouse 1:1000; Millipore MAB1637, Germany), glutamine synthetase (GS; Mouse 1:500; Millipore MAB302, Germany), ionized calcium binding adapter molecule 1 (IBA-1; Goat, 1:500; Abcam ab5076, UK), 200 kDa neurofilament protein (NF200; Mouse, 1:1000; Millipore, MAB5266, Germany), calcitonin gene-related peptide (CGRP; Goat, 1:3000; Bio-Rad 1720-9007, USA), isolectin B4 (IB4; 1:50; Sigma L2895, USA), NF-κB p65 (Rabbit, 1:1000; CST, 8242S, USA), Phospho-NF-κB p65 (Rabbit, 1:1000; CST, 3033S, USA) and SOX11 (Rabbit, 1:50; MCE HY-P80332, USA). The sections were then incubated for 2 h at room temperature with Cy3‐ or Alexa 488‐conjugated secondary antibodies (1:1000, Jackson ImmunoResearch, 715-167-003, 715-547-003, and 106-546-003, USA). Fluorescence images were acquired using a Leica SP8 confocal microscope (Germany). We used three mice per group, three slices of DRG for each mouse for staining, photographing, and counting positive neurons.

### Cell Culture and Transfection

Neuro-2a and HEK293T cells were purchased from the Cell Bank of the Chinese Academy of Sciences (TCM29 and SCSP-502, Shanghai, China) and cultured in Dulbecco’s Modification of Eagle’s Medium (DMEM, Corning, 10-013-CV, USA) supplemented with 4.5 g/L glucose, L-glutamine, and sodium pyruvate, along with 10% fetal bovine serum (FBS, Gibco, 10099141C, USA) and 1% penicillin/streptomycin (Gibco, 15140122, USA). Cells were incubated at 37 °C in a humidified atmosphere containing 5% CO_2_. For transfection, Lipofectamine 3000 (Invitrogen, L3000015, USA) was used according to the manufacturer’s instructions.

### Bioinformatics Analysis

Cell subtype-specific expression profiling of cerebellins was performed in the web tool of spatial transcriptomics data of single DRG neurons from eight organ donors (sensoryomics.com) [4]. The GSE24982 data set acquired from the Gene Expression Omnibus (GEO) database was used to detect differentially expressed genes in DRG after SNL in rats [22]. The SOX11 recognition sites in the *Cbln2* promoter were predicted using Jaspar software [23] with a relative profile score threshold of 80%.

#### Dual-luciferase Reporter Assay

The 2000 bp-length fragment upstream of the *Cbln2* gene was inserted into pGL3-Basic Vector (Promega, USA, E1751) and verified by sequencing (GENCEFE Biotech, Wuxi, China) after transformation of DH5α competent cells (Tiangen, Beijing, China, CB101-02). The full-length of the *Sox11* ORF was inserted into the pcDNA3.1 Vector and verified by sequencing after transformation of DH5α competent cells (GENCEFE Biotech, Wuxi, China).

For the luciferase reporter assay, treatment and control plasmids were transfected into HEK293T cells along with the pRL-TK plasmid (Promega, USA, E2241) as an internal control. Fluorescence was measured after 48 h following the instructions of Dual-Luciferase® Reporter Assay System (Promega, USA, E1960). In brief, cells were washed once with PBS and lysed with Passive Lysis Buffer under rotary shaking at room temperature for 15 min. Lysates were centrifuged at 12,000 r/min for 10 min, and 100–120 μL supernatants were transferred to new tubes. A 20 μL aliquot of each supernatant was loaded into an opaque white 96-well plate. Firefly luciferase activity was quantified immediately after adding 50 μL Luciferase Assay Reagent II. Renilla luciferase activity was measured within 18 s following the addition of 50 μL Stop & Glo Reagent. Activities of firefly and renilla luciferase were measured using a Synergy 2 multidetection microplate reader (BioTek, USA).

#### ChIP-PCR

According to the manufacturer's instructions, the Chromatin IP Kit (CST, Cat#25268, Danvers, MA, USA) was used to carry out a chromatin immunoprecipitation (ChIP) assay on DRG tissues. In brief, the mouse DRG tissue was processed in an enzyme-free tube with PBS buffer and a protease inhibitor for homogenization. Homogenates underwent cross-linking with 37% formaldehyde for 10 min at room temperature, followed by de-crosslinking with a 1.25 mol/L glycine solution on ice for 5 min. After centrifuging at low temperatures, the DNA–protein complexes were collected using sonication, PBS buffer washing, and WLB solution addition. The complexes of DNA and protein were incubated in the wells with antiacetyl H3, IgG, and SOX11 antibodies (MCE HY-P80332, USA) at 4 °C overnight.

Once incubation was complete, the wells were cleaned with WB solution, and RNase A solution was added, followed by a 30 min reaction at 42 °C. Protein kinase K was then added, and the reaction proceeded for 4 h at 60 °C. After purification in the wells, the DNA was transferred to EP tubes without enzymes and heated in a metal pot at 60 °C for 30 min. PCR amplification was executed, then samples underwent electrophoresis on a 2% agarose gel, followed by chemiluminescence imaging. The DNA fractions obtained through immunoprecipitation were analyzed using qRT-PCR with primers specific to mouse *Cbln2*. The following primers were used: Forward 5′-CCT TCT ACA GAA TTG CAG CCA G-3′, Reverse 5′-TCC CGA GAG CTA TCC TCA TTC-3′.

#### RNA-seq

DRG tissues were harvested 6 h post-intrathecal injection of CBLN2 or PBS (*n* *= *3/group) for transcriptome profiling. Total RNA was isolated using TRIzol reagent (Invitrogen, USA) followed by DNase I treatment (Takara, Japan) to eliminate genomic DNA contamination. RNA integrity was confirmed using Agilent 2100 Bioanalyzer (RNA integrity number > 8.0) and quantified via NanoDrop 2000 spectrophotometer (Thermo Scientific, USA). Libraries were constructed with 1.5 μg total RNA using the NEBNext Ultra RNA Library Prep Kit (Illumina, USA) according to the manufacturer's protocol. Briefly, polyadenylated mRNA was enriched by oligo (dT) magnetic beads and fragmented in 5X NEBNext First Strand Synthesis Buffer. Double-stranded cDNA was synthesized using random hexamer priming (M-MuLV Reverse Transcriptase) and subsequent DNA Polymerase I digestion. After end repair and adenylation, adaptors were ligated, followed by AMPure XP bead purification (Beckman Coulter, USA) to select 200–250 bp fragments. PCR amplification was performed with Phusion High-Fidelity DNA Polymerase using index primers (12 cycles). Library quality was validated by Agilent 2100 Bioanalyzer prior to 150 bp paired-end sequencing on the Illumina NovaSeq 6000 platform (Allwegene Tech, China).

Raw reads were obtained using bcl2fastq and preprocessed by removing adaptor sequences and low-quality bases (Q < 20) using FastQC [24]. Clean reads were aligned to the GRCm39 reference genome using Hisat2 [25] with default parameters. Gene-level counts were quantified via featureCounts [26] and StringTie [27], followed by FPKM (fragments per kilobase of exon model per million mapped fragments) normalization for expression quantification. Differential expression analysis was conducted using limma [28] with Benjamini-Hochberg correction (|log2 FC| > 1 and adjusted *P* < 0.05). KEGG pathway enrichment was analyzed through KOBAS (v3.0) with Fisher's exact test (*P* < 0.05).

#### Whole-cell Patch-clamp

Lumbar DRG (L4–L6) neurons were dissected from ICR mice (4–6 weeks), digested with collagenase I (3.54 mg/mL, Gibco, USA) and Dispase II (1.65 mg/mL, Roche, Switzerland), and cultured in Neurobasal medium (Gibco, 10888022, USA) in regular 95% air and 5% CO_2_ at 37 °C.

Whole-cell patch-clamp recordings were performed on small-diameter DRG neurons (< 25 μm), including neuronal excitability, action potential (AP) firing frequency, and resting membrane potential level. Glass microelectrodes (resistance 4–8 MΩ) were fabricated with a P-97 flaming micropipette puller (Sutter Instruments, USA). Neuronal excitability was assessed via ramp and square-pulse current injections (100–300 pA). Data were analyzed using pClamp10 software (Axon Instruments, USA).

#### Quantification and Statistics

Data are expressed as mean ± SEM. The behavioral data were analyzed by two-way repeated measures (RM) ANOVA followed by Bonferroni’s test. The qRT-PCR data were analyzed by one-way ANOVA followed by Bonferroni’s test. For western blot, the density of specific bands was measured with Image J (NIH, USA). Differences between the two groups were compared using Student’s t-test. GraphPad Prism v8.0 was used for statistical analyses. Significance thresholds were defined as follows: *P < 0.05, **P < 0.01, and ***P < 0.001.

## Results

### CBLN2 Exists in Proprioceptive and Nociceptive Neurons of Humans and Mice

**Fig. 1:**
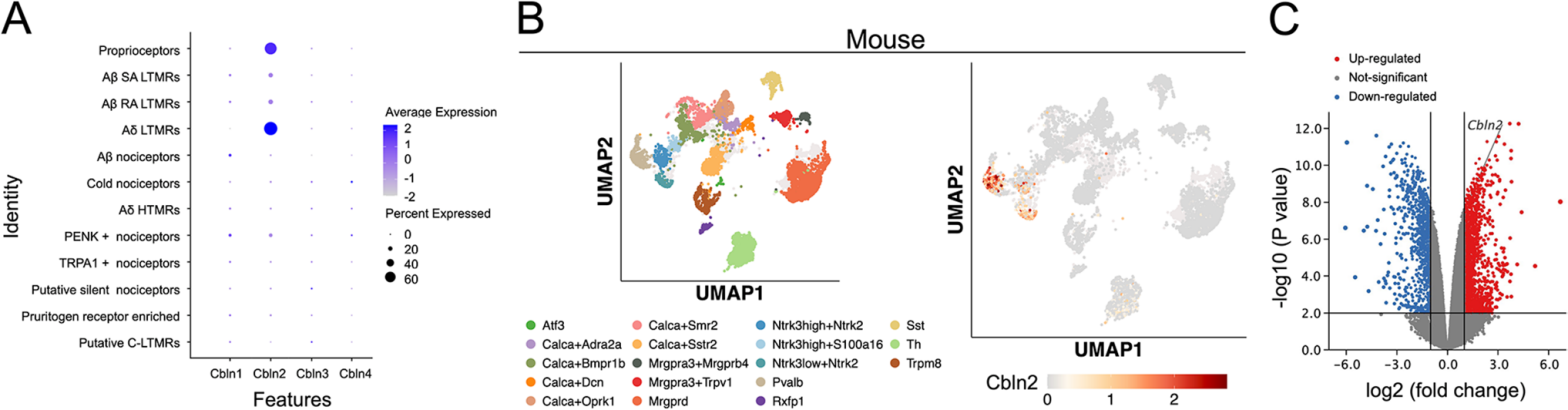
CBLN2 exists in proprioceptive and nociceptive neurons of humans and mice**. A** Analysis of spatial transcriptomic data from human DRG sensory neurons. CBLN2 was predominantly expressed in Aδ LTMRs, proprioceptors, and Aβ LTMRs among 12 human sensory neuron clusters, with minimal expression of other cerebellins. **B** Analysis of CBLN2 expression patterns in the harmonized DRG atlas of mice. Ntrk3low + Ntrk2 (Aδ-LTMRs, pctExpress = 89.0%), Pvalb (proprioceptors, pctExpress = 66.9%), and Ntrk3high + Ntrk2 (Aβ-LTMRs, pctExpress = 36.3%). **C** Volcano plot of RNA-seq data from GSE24982. *Cbln2* was significantly increased in rat L5 DRG at 4 weeks post-SNL (log2 FC = 1.1, *P =* 1.7e-08).

We retrieved the expression profile of cerebellins in the spatial transcriptomic data from human DRG sensory neurons (sensoryomics.com) [4]. The result revealed that CBLN2 was predominantly expressed in Aδ low threshold mechanoreceptors (LTMRs), proprioceptors, and Aβ LTMRs among 12 human sensory neuron clusters, with minimal expression of other cerebellins (Fig. 1A). Analysis of the harmonized DRG atlas (painseq.shinyapps.io/harmonized_painseq_v1) [5] showed conserved CBLN2 expression patterns across species. In mice, it localized to Ntrk3low + Ntrk2 (Aδ-LTMRs, pctExpress = 89.0%), Pvalb (proprioceptors, pctExpress = 66.9%), and Ntrk3high + Ntrk2 (Aβ-LTMRs, pctExpress = 36.3%) neurons (Fig. 1B).

As the most common neuropathic pain model, SNL induced upregulation of CBLN2 in L5 DRG of rats (GSE24982) [22]. *Cbln2* expression significantly increased at 4 weeks post-SNL (log2 FC = 1.1, *P = *1.7e-08, Fig. 1C). Combined with the previously predicted expression profile, we speculated that CBLN2 in DRG may be involved in the regulation of pain.

### SNL Increases CBLN2 Expression in DRG Neurons

**Fig. 2:**
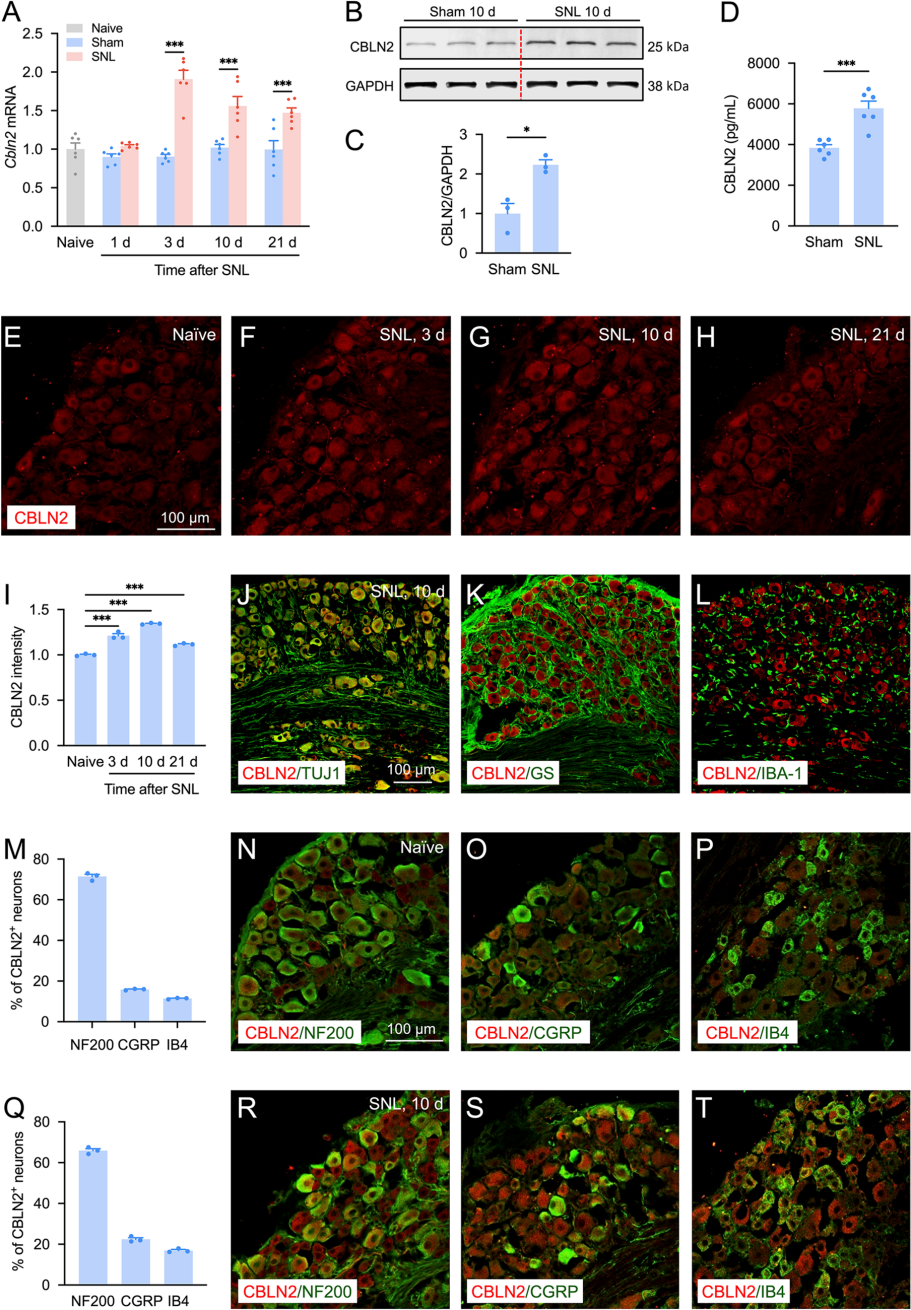
SNL increases CBLN2 expression in DRG neurons. **A** *Cbln2* mRNA expression in DRG of naïve, sham-, and SNL-operated mice. ***P < 0.001 vs. corresponding sham group, two-way ANOVA followed by Bonferroni’s test, *n =* 6 mice/group. **B–C** Western blot analysis showed increased CBLN2 protein in DRG at 10 days post-SNL. *P < 0.05 vs. sham, Student’s *t*-test, *n =* 3 mice/group. **D** ELISA analysis showed CBLN2 protein upregulation at day 10 post-SNL. ***P < 0.001 vs. sham, Student’s *t*-test, *n =* 6 mice/group.** E–I** Immunofluorescence of CBLN2 in the DRG. ***P < 0.001, one-way ANOVA followed by Bonferroni’s test, *n =* 3 mice/group. **J–L** Immunofluorescence double-staining of CBLN2 with TUJ1 (**J**), GS (**K**), and IBA-1 (**L**) in the DRG 10 days after SNL. **M–P** Immunofluorescence double-staining of CBLN2 with NF200 (**N**), CGRP (**O**), and IB4 (**P**) in the DRG of naïve mouse. **Q–T** Immunofluorescence double-staining of CBLN2 with NF200 (**R**), CGRP (**S**), and IB4 (**T**) in the DRG at 10 d post-SNL.

Our temporal analysis via qRT-PCR showed that *Cbln2* mRNA peaked at 3 days post-SNL in the mice (1.9-fold vs. sham), remaining elevated at 10 days (1.6-fold) and 21 days (1.5-fold, Fig. 2A). Western blot confirmed a 2.2-fold increase in CBLN2 protein at 10 days post-SNL (Fig. 2B–C). ELISA showed a similar CBLN2 upregulation at day 10 (1.5 ± 0.3-fold vs. sham, Fig. 2D). Immunofluorescence revealed progressive CBLN2 upregulation: 1.2-fold at 3 days, 1.3-fold at 10 days, and 1.1-fold at 21 days compared to naïve mice (Fig. 2E–I).

Further immunofluorescence double-staining confirmed CBLN2 expression in TUJ1^+^ neurons but not in GS^+^ satellite glia or IBA-1^+^ macrophages at 10 days post-SNL (Fig. 2J–L). In addition, 71.4% of CBLN2^+^ neurons co-localized with NF200 (large and medium-sized myelinated neurons), 15.9% with CGRP (peptidergic nociceptors), and 11.4% with IB4 (non-peptidergic nociceptors) in the DRG of naïve mice (Fig. 2M–P). Ten days after SNL, 65.9% of CBLN2^+^ neurons co-localized with NF200, 22.4% with CGRP, and 16.9% with IB4 (Fig. 2Q–T), showing an increase of CBLN2 in small-diameter nociceptors. These results indicate that CBLN2 may play a role in SNL-induced neuropathic pain.

### SOX11 Regulates the Transcription of *Cbln2*

**Fig. 3:**
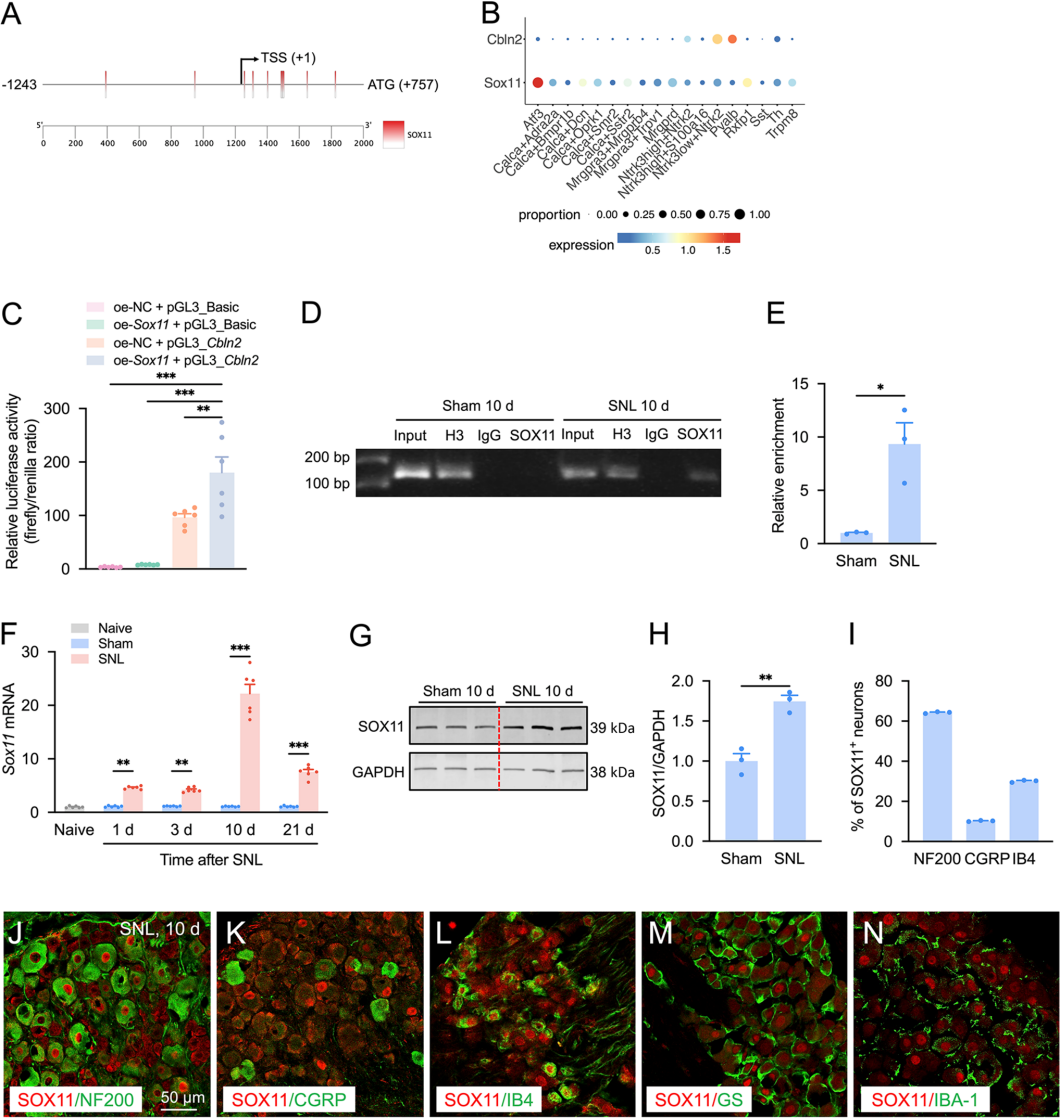
SOX11 regulates the transcription of *Cbln2*. **A** A *cis*-regulatory element analysis of the 2000-bp sequence (−1243 to +757) upstream of the *Cbln2* translation initiation site using JASPAR software predicted 12 putative SOX11 binding sites. **B** Analysis of SOX11 and CBLN2 expression patterns in the harmonized DRG neuronal reference atlas. The two genes were co-expressed in the Ntrk3low + Ntrk2, Pvalb, and Ntrk3high + Ntrk2 neurons. **C** Dual-luciferase reporter assays demonstrated that SOX11 overexpression significantly enhanced *Cbln2* promoter-driven reporter gene activity. **D–E** Representative blots and summarized ChIP-qPCR data demonstrate alterations in the binding of SOX11 with *Cbln2* following SNL. Input, total purified fragments. Student’s unpaired *t*-test. Data are mean ± SEM of biological replicates *n =* 3, *P < 0.05 versus sham group. **F** *Sox11* mRNA expression in DRG of naïve, sham-, and SNL-operated mice. **P < 0.01, ***P < 0.001 vs. corresponding sham group, two-way ANOVA followed by Bonferroni’s test, *n =* 6 mice/group. **G–H** Western blot analysis showed increased SOX11 protein in DRG at 10 d post-SNL. **P < 0.01 vs. sham, Student’s *t*-test, *n =* 3 mice/group. **I** Quantification of SOX11 co-localization with NF200, CGRP, and IB4 at 10 d post-SNL. **J–N** Immunofluorescence double-staining of SOX11/NF200 (J), SOX11/CGRP (K), SOX11/IB4 (L), SOX11/GS (M), and SOX11/IBA-1 (N) in the DRG at 10 d post-SNL.

Given the critical role of transcription factors in gene expression regulation [29], we investigated the molecular mechanism underlying CBLN2 upregulation after SNL. A *cis*-regulatory element analysis of the 2000-bp sequence (− 1243 to + 757) upstream of the *Cbln2* translation initiation site using JASPAR software predicted 12 putative SOX11 binding sites (Fig. 3A). Furthermore, analysis of the harmonized DRG neuronal reference atlas [5] revealed that SOX11 was co-expressed in the three neuronal subtypes with the highest CBLN2 expression: Ntrk3low + Ntrk2 (Aδ-LTMRs, pctExpress = 52.9%), Pvalb (proprioceptors, pctExpress = 20.8%), and Ntrk3high + Ntrk2 (Aβ-LTMRs, pctExpress = 29.2%) neurons (Fig. 3B). Dual-luciferase reporter assays demonstrated that SOX11 overexpression significantly enhanced *Cbln2* promoter-driven reporter gene activity (Fig. 3C). Consistently, in mouse DRG lysates, ChIP-PCR analysis demonstrated amplifiable fragments within the *Cbln2* gene promoter from the immunoprecipitation complex with anti-SOX11 antibodies in sham-operated mouse, affirming the targeted interaction of SOX11 with the *Cbln2* gene. Notably, SNL contributed to a concomitant increase in the binding affinity of SOX11, as reflected by band density, within the ipsilateral DRG on SNL 10 d in comparison to the levels observed in sham-operated rats (Fig. 3D). ChIP-qPCR result showed that SNL increased the binding affinity of SOX11 to the *Cbln2* promoter fragment (9.2 ± 2.9-fold vs sham, Fig. 3E). These findings suggest that SOX11 positively regulates *Cbln2* transcription.

To evaluate *Sox11* dynamics in neuropathic pain, we quantified its mRNA levels in DRG at multiple post-SNL time points. qRT-PCR showed significant upregulation of *Sox11* mRNA at day 1, 3, 10, and 21 after SNL compared to sham controls, peaking at day 10 (20.3-fold increase, Fig. 3F). Western blot confirmed a 1.8-fold elevation in SOX11 protein levels at day 10 (Fig. 3G–H). At 10 days after SNL, 64.3% of SOX11^+^ cells co-expressed NF200, 10.2% co-expressed CGRP, and 30.1% co-expressed IB4, but minimal overlap with GS^+^ or IBA-1^+^ cells (Fig. 3I–N), indicating SOX11/CBLN2 co-localization in large and medium-diameter, peptidergic, and non-peptidergic neurons.

### SOX11/CBLN2 Contributes to SNL-induced Mechanical Allodynia and Thermal Hyperalgesia

**Fig. 4:**
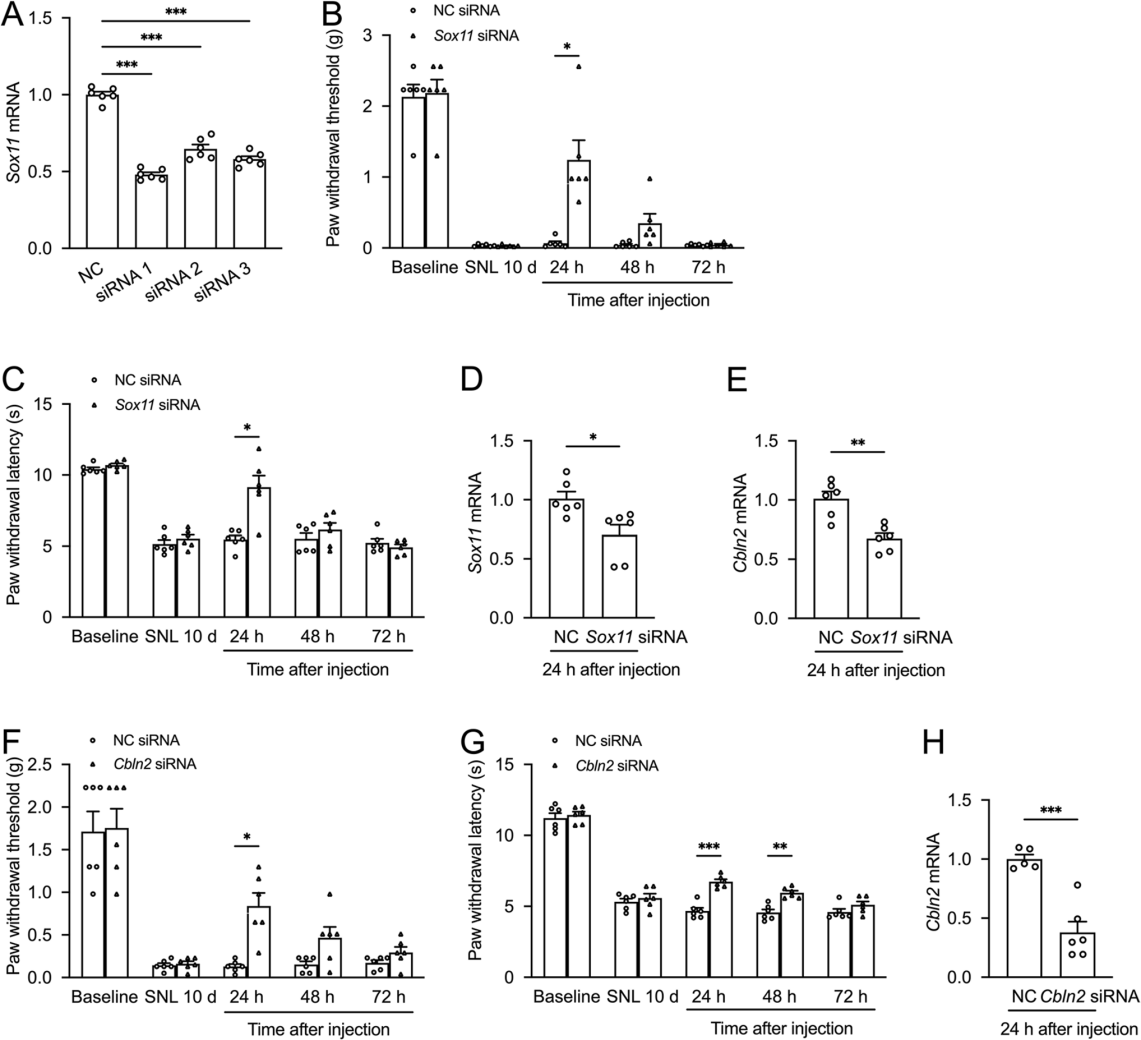
SOX11 and CBLN2 contribute to SNL-induced mechanical allodynia and thermal hyperalgesia. **A** Validation of *Sox11*-targeting siRNA knockdown efficiency in Neuro-2a cells. siRNA-1, siRNA-2 and siRNA-3 significantly reduced *Sox11* expression compared to the negative control. ***P < 0.001 vs. NC, one-way ANOVA followed by Bonferroni’s test, *n =* 6 wells/group. **B–C** Intrathecal delivery of *Sox11* siRNA1 at 10 d post-SNL alleviated mechanical allodynia and thermal hyperalgesia 24 h post-injection. *P < 0.05 vs. NC siRNA; two-way ANOVA followed by Bonferroni’s test, *n =* 6 wells/group. **D–E** *Sox11* siRNA-1 reduced *Sox11* and *Cbln2* expression. *P < 0.05, **P < 0.01, Student’s *t-*test, *n =* 6 mice/group. **F–G** Intrathecal delivery of *Cbln2* siRNA at 10 d post-SNL alleviated mechanical allodynia 24 h post-injection and thermal hyperalgesia 24–48 h post-injection. *P < 0.05, **P < 0.01, ***P < 0.001 vs. NC, two-way ANOVA followed by Bonferroni’s test, *n =* 6 wells/group. **H** *Cbln2* siRNA reduced *Cbln2* expression. ***P < 0.001, Student’s *t*-test, *n =* 6 mice/group.

To assess functional roles, we screened three *Sox11*-targeting siRNAs in Neuro-2a cells, with siRNA1 achieving maximal knockdown (48.1% reduction, Fig. 4A). Intrathecal delivery of siRNA1 at day 10 alleviated mechanical allodynia and thermal hyperalgesia 24 h post-injection (Fig. 4B–C). At the same time, *Sox11* siRNA reduced *Sox11* and *Cbln2* mRNA levels to 69.7% and 66.9%, respectively (Fig. 4D–E). Similarly, *Cbln2* siRNA attenuated mechanical allodynia at 24 h post-injection and thermal hyperalgesia at 24–48 h post-injection and reduced *Cbln2* expression to 37.9% (Fig. 4F–H). These results implicate SOX11-mediated transcriptional regulation of *Cbln2* in neuropathic pain.

### CBLN2 Induces Pain Hypersensitivity and the Upregulation of Multiple Chemokines/Receptors in the DRG

**Fig. 5:**
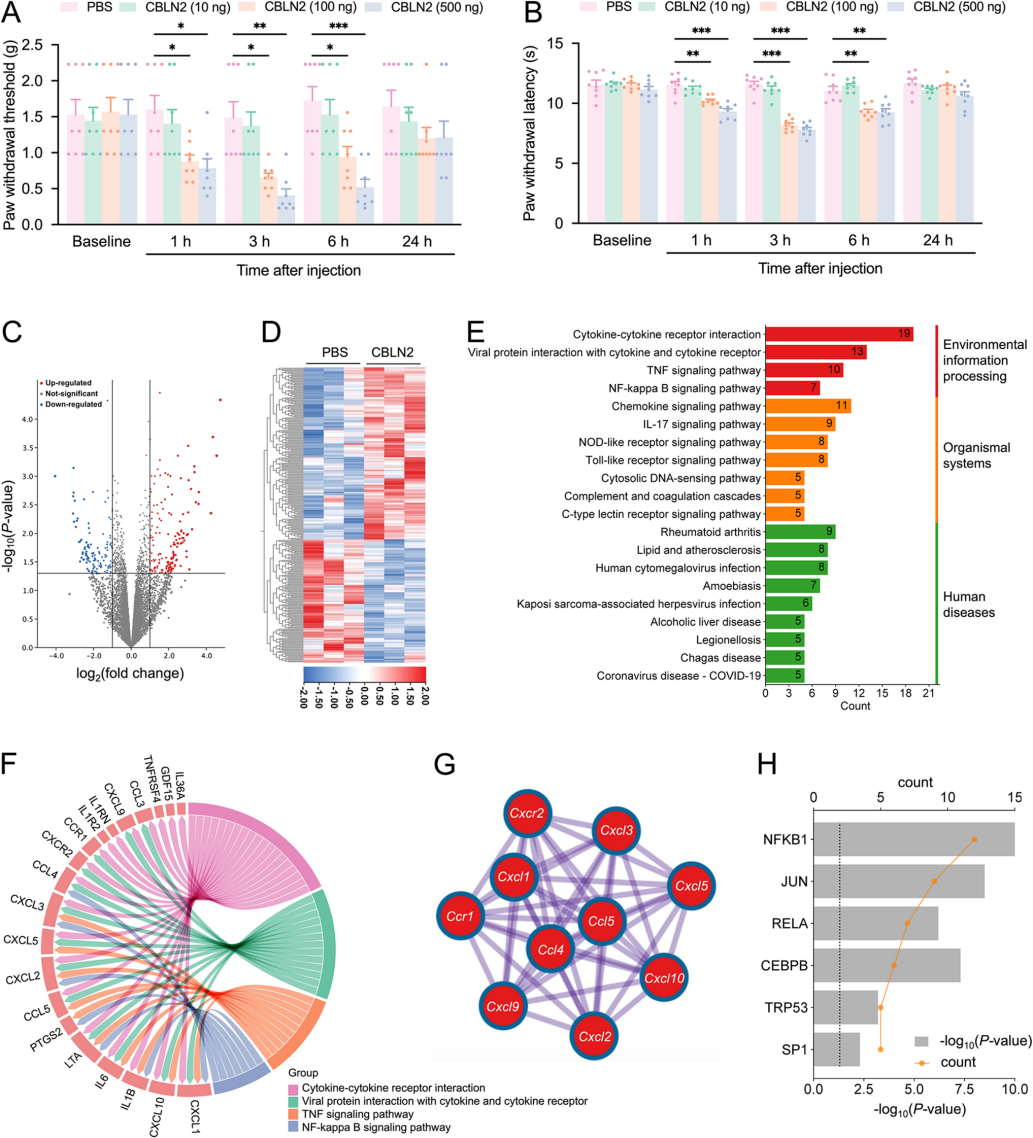
CBLN2 induces pain hypersensitivity and gene expression changes in the DRG.** A–B** Intrathecal injection of CBLN2 induced mechanical allodynia and thermal hyperalgesia at 1-6 h post-injection. *P < 0.05, **P < 0.01, ***P < 0.001, two-way RM ANOVA followed by Bonferroni’s test, *n =* 8 mice/group. **C** Volcano plot of differentially expressed genes in DRG transcriptome sequencing after intrathecal injection of CBLN2. Among them, there were 163 up-regulated genes and 116 down-regulated genes. Differentially expressed gene screening criteria: |log2 FC| ≥ 1 and adjusted *P* ≤ 0.05. Red dots represented up-regulated genes, blue dots represented down-regulated genes, and gray dots represented genes with no significant difference. **D** Heat map of differentially expressed genes. **E** KEGG analysis of differentially expressed genes. *P <* 0.01, Count ≥ 5. **F** Genes included in the four pathways respond to environmental stimuli. **G** Schematic diagram of the interaction of up-regulated chemokines. **H** Transcription factor analysis of up-regulated genes. Twelve upregulated genes were regulated by NFKB1, *P <* 0.01, Count ≥ 5.

To further elucidate the role of CBLN2 in neuropathic pain, intrathecal administration of CBLN2 recombinant protein at varying doses was performed. Behavioral assessments revealed that 100 ng and 500 ng CBLN2 induced mechanical allodynia and thermal hyperalgesia at 1 h post-injection, persisting up to 6 h compared to PBS controls. In contrast, 10 ng CBLN2 failed to evoke hypersensitivity behaviors (Fig. 5A, B).

To investigate downstream molecular mechanisms, high-throughput sequencing of DRG tissues was conducted at 6 h post-intrathecal injection of 100 ng CBLN2. Among 16,661 detected genes, 163 were upregulated and 116 were downregulated (|log2 FC| ≥ 1, adjusted *P* ≤ 0.05) relative to PBS controls (Fig. 5C, D). KEGG enrichment analysis (*P <* 0.01, count ≥ 5) categorized upregulated genes into three classes: environmental information processing, biological systems, and human diseases (Fig. 5E). Key pathways included cytokine-cytokine receptor interactions, TNF signaling, and NF-κB activation (Fig. 5F). Chord diagrams highlighted ten chemokines/receptors (*Cxcr2*, *Cxcl1*, *Ccr1*, *Cxcl9*, *Ccl4*, *Cxcl3*, *Ccl5*, *Cxcl2*, *Cxcl5*, *Cxcl10*) forming a protein-protein interaction cluster (STRING physical score > 0.132, *P <* 1E-5; Fig. 5G). TRRUST (version 2) [30] predicted NF-κB as a transcriptional regulator for 12 upregulated genes (*Cd80*, *Cxcl1*, *Cxcl10*, *Il-1b*, *Il-6*, *Plaur*, *Ptgs2*, *Ccl5*, *Cxcl2*, *Tnfrsf4*, *Upp1*, *Trem1*; adjusted P ≤ 0.01, count ≥ 5; Fig. 5H), suggesting that CBLN2 may modulate neuropathic pain via NF-κB signaling.

### NF-κB Mediates CBLN2- and SNL-Induced Pain Hypersensitivity

**Fig. 6:**
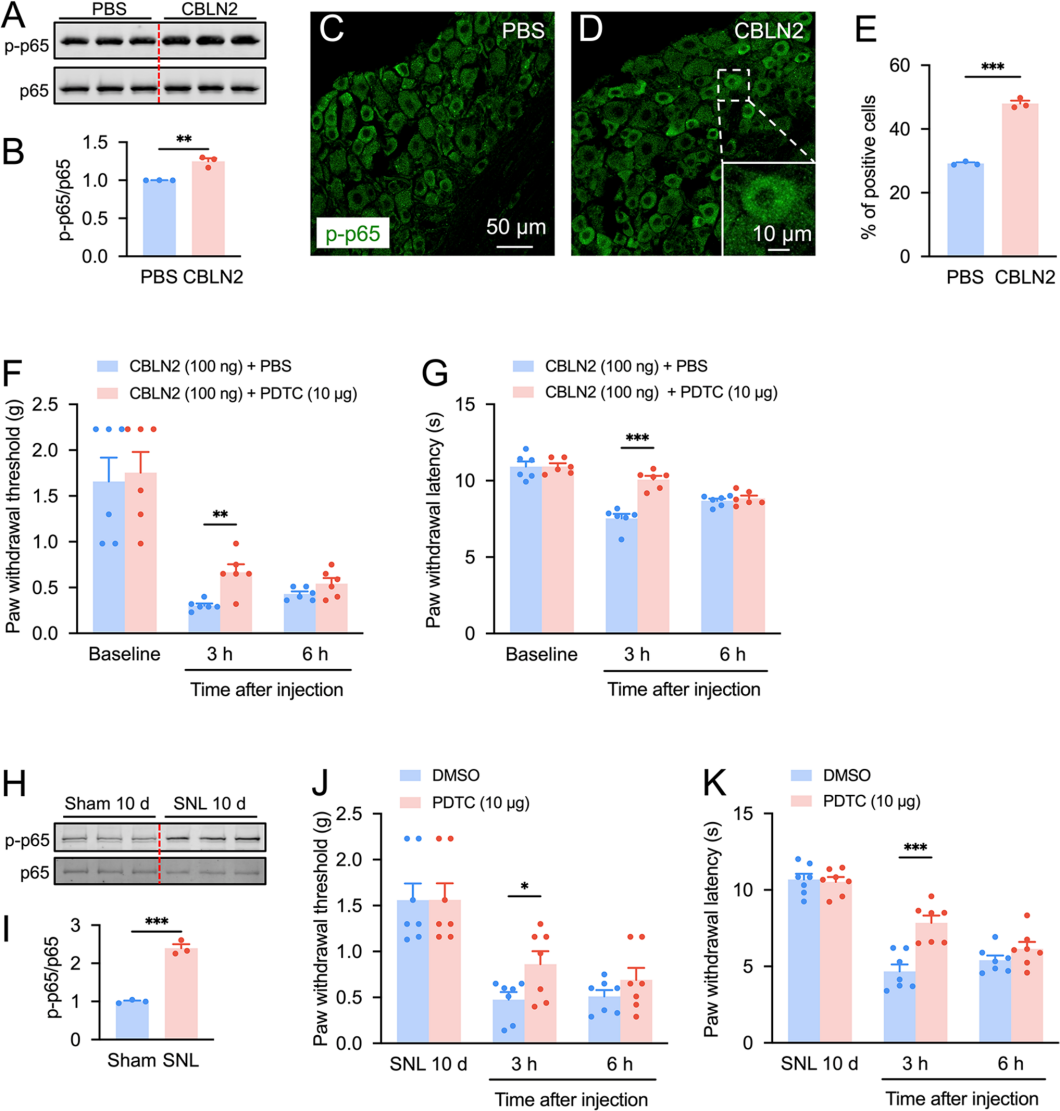
NF-κB inhibitor attenuates CBLN2-induced mechanical allodynia and thermal hyperalgesia.** A–B** Western blot showed that intrathecal injection of CBLN2 induced p-p65 in the DRG. **P < 0.01, Student’s *t*-test, *n =* 3 mice/group. **C–E** Immunostaining showed p65 activation in the DRG after intrathecal CBLN2. ***P < 0.001, Student’s *t*-test, *n =* 3 mice/group. **F–G** Intrathecal injection of PDTC attenuated CBLN2-induced mechanical allodynia and thermal hyperalgesia at 3 h post-injection. **P < 0.01, ***P < 0.001 vs. PBS; two-way ANOVA followed by Bonferroni’s test, *n =* 6 wells/group. **H–I** SNL induced p-p65 upregulation in the DRG. **P < 0.01, Student’s *t*-test, *n =* 3 mice/group. **J–K** Intrathecal injection of PDTC attenuated SNL-induced mechanical allodynia and thermal hyperalgesia at 3 h post-injection. *P < 0.05, ***P < 0.001 vs. DMSO; two-way ANOVA followed by Bonferroni’s test, *n =* 7 wells/group.

Next, we investigated whether NF-κB is a downstream kinase of CBLN2. Western blot analysis demonstrated a 1.3-fold elevation in phosphorylated p65 (p-p65) levels in DRG tissues of CBLN2-injected mice versus controls (Fig. 6A, B). Immunofluorescence confirmed the 1.6-fold upregulation of p-p65 and showed its distribution in both the cytoplasm and nucleus, indicating potential nuclear translocation (Fig. 6C–E). Furthermore, pretreatment with the NF-κB inhibitor PDTC attenuated CBLN2-induced mechanical allodynia and thermal hyperalgesia at 3 h post-injection (Fig. 6F, G). Similarly, SNL elevated p-p65 levels (2.4-fold vs. sham; Fig. 6H, I) on day 10, and PDTC alleviated SN-induced pain hypersensitivity behaviors at 3 h post-injection (Fig. 6J, K). These findings indicate that NF-κB mediates the role of CBLN2 in the pathogenesis of neuropathic pain.

### *Sox11* siRNA and NF-κB Inhibitor Significantly Attenuate CBLN2-induced Upregulation of Proinflammatory Cytokines

**Fig. 7:**
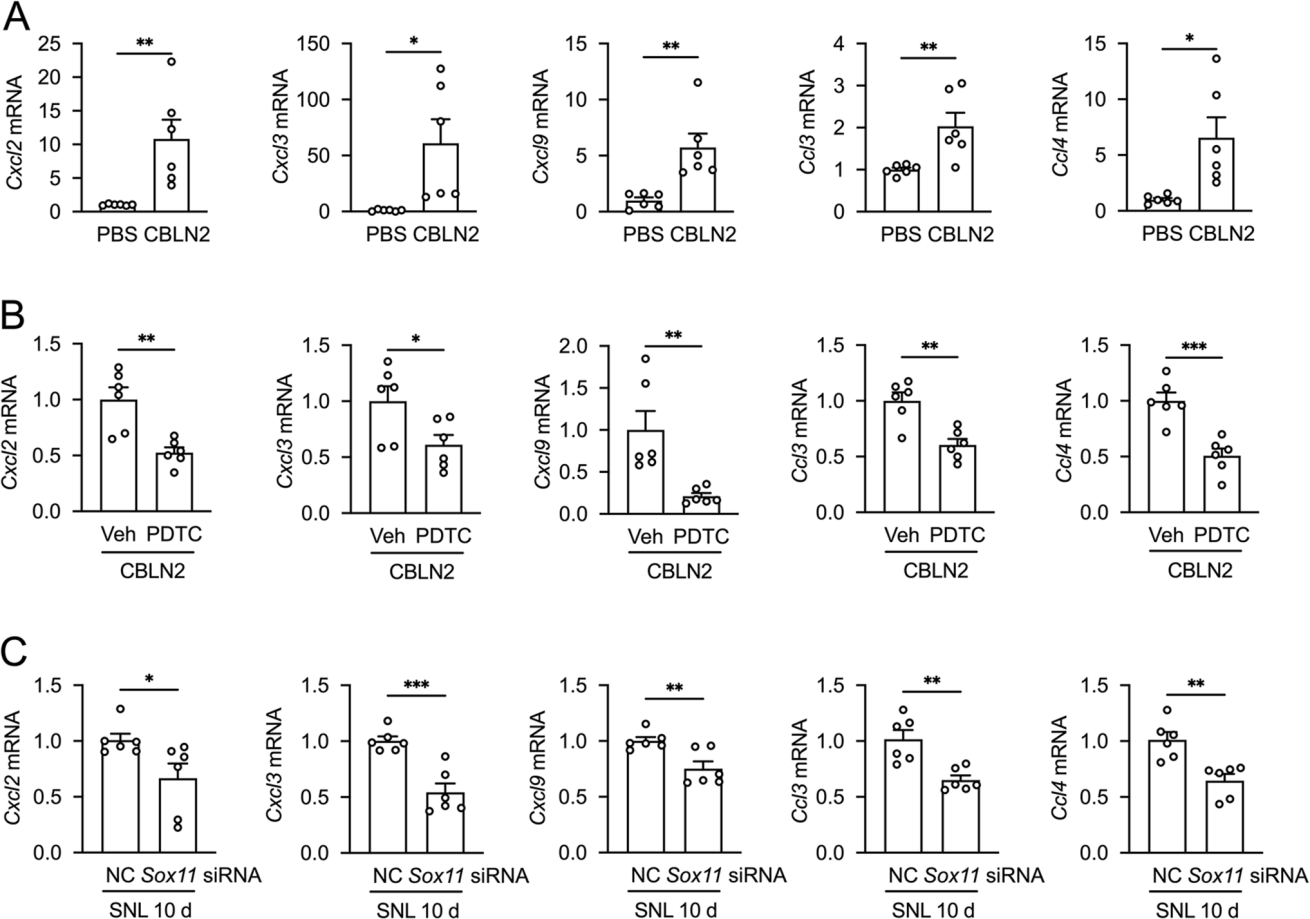
NF-κB inhibitor and *Sox11* siRNA decreased CBLN2-induced proinflammatory cytokines upregulation. **A** Intrathecal administration of CBLN2 upregulated mRNA levels of *Cxcl2*, *Cxcl3*, *Cxcl9*, *Ccl3*, and *Ccl4* at 6 h post-CBLN2 administration. *P < 0.05, **P < 0.01 vs. PBS, Student's *t*-test, *n =* 6 wells/group. **B** Pretreatment with PDTC reduced CBLN2-induced chemokine expression. *P < 0.05, **P < 0.01, ***P < 0.001 vs. vehicle, Student's *t*-test, *n =* 6 wells/group. **C** Intrathecal delivery of *Sox11*-targeting siRNA reduced the expression of these chemokines. *P < 0.05, **P < 0.01, ***P < 0.001 vs. NC, Student's *t*-test, *n =* 6 wells/group.

As chemokine-encoding genes constituted the predominant category among both core pathway components and transcriptionally regulated downstream targets after CBLN2 treatment (Fig. 5F–H), and protein-protein interaction network analysis (STRING database) demonstrated significant clustering of these chemokines (Fig. 5G), we performed systematic expression profiling of these NF-κB-associated chemokines. qRT-PCR analysis demonstrated increased mRNA levels of *Cxcl2*, *Cxcl3*, *Cxcl9*, *Ccl3*, and *Ccl4* at 6 h post-CBLN2 administration (Fig. 7A). In addition, pretreatment with the NF-κB inhibitor PDTC markedly reduced CBLN2-induced chemokine expression (Fig. 7B). Notably, intrathecal delivery of *Sox11*-targeting siRNA at 10 d post-SNL significantly reduced the levels of these chemokines (Fig. 7C). These results indicate that SOX11/CBLN2/NF-κB signaling pathway regulates downstream proinflammatory cytokines.

### NF-κB Inhibitor Decreases CBLN2-induced Hyperexcitability of DRG Neurons

**Fig. 8:**
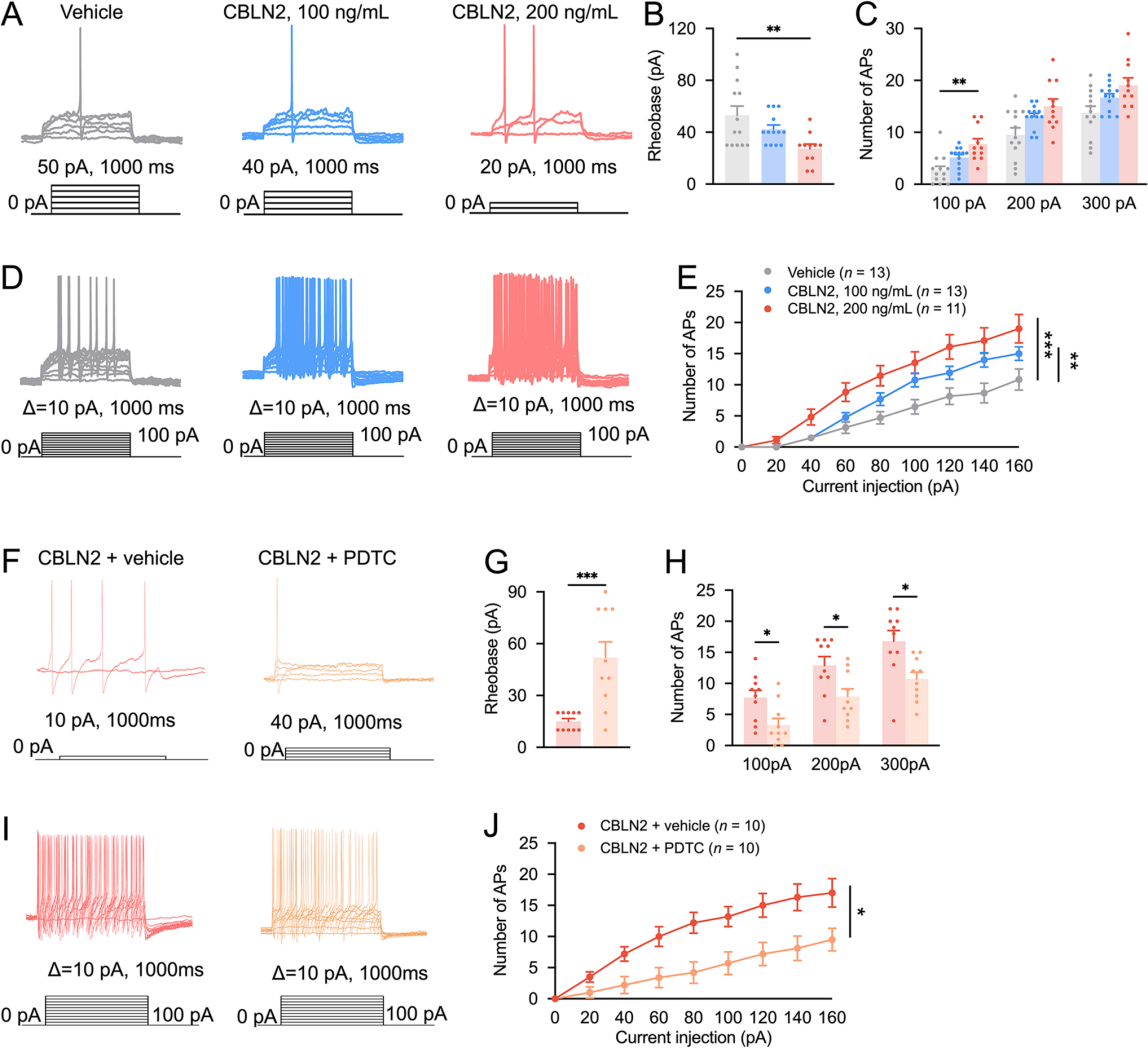
CBLN2-induced increase of the APs in DRG neurons is blocked by NFkB inhibitor. **A** Representative traces of AP evoked by rheobase current in vehicle- and CBLN2-treated (100, 200 ng/mL) DRG neurons. **B** Incubation with 200 ng/mL CBLN2 decreased the rheobase current required for AP generation of DRG neurons. **P < 0.01, one-way ANOVA followed by the Bonferroni’s test, *n =* 11-13 neurons/group from 3 to 4 mice. **C** Incubating with 200 ng/mL CBLN2 increased the number of evoked AP of DRG neurons when stimulated by 100 pA ramp current. **P < 0.01, two-way RM ANOVA followed by Bonferroni’s test, *n = *11–13 neurons/group from 3 to 4 mice. **D** Representative traces of AP evoked by depolarizing current steps in vehicle- and CBLN2-treated (100, 200 ng/mL) DRG neurons. **E** CBLN2 increased the number of APs in response to 160 pA, 1000 ms current injection. **P < 0.01, ***P < 0.001, two-way RM ANOVA followed by Bonferroni’s test, *n =* 11-13 neurons/group from 3-4 mice. **F** Representative traces of AP evoked by rheobase current in CBLN2-treated (200 ng/mL) and PDTC-pretreated (50 μmol/L) DRG neurons. **G** PDTC increased the rheobase current required for AP generation. ***P < 0.001, Student’s *t*-test, *n =* 10 neurons/group from 3-4 mice. **H** PDTC decreased the AP firing frequency. *P < 0.05, two-way RM ANOVA followed by Bonferroni’s test, *n =* 10 neurons/group from 3-4 mice. **I** Representative traces of AP evoked by depolarizing current steps in CBLN2-treated (200 ng/mL) and PDTC-pretreated (50 μmol/L) DRG neurons. **J** PDTC attenuated the number of APs in response to 160 pA, 1000 ms current injection. *P < 0.05, two-way RM ANOVA followed by Bonferroni’s test, *n* = 10 neurons/group from 3 to 4 mice.

Increased excitability of DRG neurons induced by peripheral nerve injury is closely correlated with neuropathic pain [31, 32]. Whole-cell patch-clamp recordings indicated that acute CBLN2 incubation (200 ng/mL, 30 min) significantly reduced the rheobase currents compared to the vehicle (Fig. 8A, B). Additionally, the number of APs was also significantly increased under the ramp current of 100 pA stimulation in CBLN2 (Fig. 8C). A series of step current (0 to 160 pA, +20 pA/step, 1000 ms) stimulation similarly elicited an elevated AP generation in CBLN2-treated neurons (Fig. 8D, E).

To further ascertain the role of NF-κB in this process, we pretreated DRG neurons with PDTC before CBLN2 incubation. The PDTC pretreatment increased the rheobase current (Fig. 8F, G). Moreover, the firing numbers also markedly decreased under a series of ramp currents (100, 200, and 300 pA) injection in neurons that were pretreated with PDTC (Fig. 8H). Step currents (0 to 160 pA, + 20 pA/step, 1000 ms) stimulation evoked APs were significantly decreased in PDTC-pretreated neurons (Fig. 8I, J). These data suggested that inhibition of NF-κB signaling mitigates CBLN2-induced neuronal hypersensitivity, highlighting the role of CBLN2-NF-κB in regulating neuron excitability associated with neuropathic pain.

## Discussion

In the present study, we demonstrated that SOX11-driven CBLN2 production in large and medium-diameter DRG neurons initiates a neuron-subtype-specific signaling cascade. As a secreted protein [33, 34], CBLN2 is likely released into the local microenvironment, where it activates NF-κB signaling in neighboring neurons, promoting neuronal hyperexcitability and chemokine production. Our findings reveal a novel SOX11-CBLN2-NF-κB axis as a key mediator of both neuronal hyperactivity and neuroinflammatory responses in SNL-induced neuropathic pain. These findings establish CBLN2 as a key regulator within the DRG neuronal microenvironment and highlight its role as an upstream mediator in the hierarchical organization of pain signaling pathways.

### CBLN2 as A Novel Molecular Mediator in Peripheral Neuropathic Pain Pathogenesis

CBLN2 exhibits evolutionarily conserved expression across species, with prominent localization in human Aδ-LTMRs, proprioceptors, and Aβ-LTMRs—a distribution pattern faithfully recapitulated in murine DRG neurons, underscoring its fundamental role in sensory processing. Its neuronal specificity and selective subtype expression suggest its involvement in myelinated mechanotransduction and nociceptive signaling, including peptidergic and non-peptidergic pathways. Notably, CBLN2 displays minimal co-expression with other cerebellin family members (CBLN1/3/4), suggesting that it may have evolved specialized roles distinct from canonical cerebellin functions.

Following SNL, CBLN2 exhibits a distinct temporal regulation pattern: mRNA levels peak at 3 days, while protein remains elevated for 21 days. This sustained upregulation contrasts with the transient induction of classical inflammatory mediators such as IL-6 or TNF-α, which typically surge in the acute phase following nerve injury [35]. The temporal dynamics of CBLN2 induction closely correlate with the progression of mechanical allodynia and thermal hyperalgesia. Moreover, the increased CBLN2 expression in small-diameter nociceptors (CGRP^+^ and IB4^+^ neurons) post-SNL suggests injury-induced plasticity, further implicating CBLN2 in the maintenance of injury-induced pain states.

Behavioral assays provide functional validation of CBLN2 as a critical mediator of neuropathic pain. Intrathecal administration of *Cbln2* siRNA alleviated SNL-induced mechanical allodynia and heat hyperalgesia, and intrathecal CBLN2 induced dose-dependent pain hypersensitivity, supporting its role as a proximal trigger of nociceptive sensitization. Moreover, the discovery of CBLN2’s pain-regulatory function significantly expands the known biological roles of cerebellins, which were previously recognized primarily for their involvement in cerebellar synaptic organization [7].

### SOX11-driven Transcriptional Regulation of CBLN2

SOX11, a transcription factor critical during development, is re-expressed in adult DRG neurons after peripheral nerve injury, where it promotes axonal regeneration, particularly in large myelinated neurons [13]. Beyond its established regenerative role, SOX11 also transcriptionally activates a broader repertoire of injury-response genes, such as *Sprr1a* [36] or *Tank* [15], suggesting it as a central regulator of injury-induced gene networks. In this study, we identified 12 high-confidence SOX11 binding sites within the *Cbln2* promoter. Their strong co-expression in Aδ-LTMRs and proprioceptors supports a direct regulatory relationship, which was further validated by dual-luciferase reporter assays demonstrating SOX11-mediated transactivation of *Cbln2*. ChIP-PCR results further confirmed that SNL enhanced the binding affinity between SOX11 and the promoter fragment of *Cbln2*. These findings provide mechanistic insight into injury-related transcriptional reprogramming and position *Cbln2*, a gene previously implicated in synaptic organization, as a novel downstream effector of SOX11.

The temporal overlap between SOX11 and CBLN2 upregulation after SNL reinforces their functional coupling. Spatial mapping revealed their co-localization in NF200^+^ neurons, consistent with a cell-autonomous mode of regulation. However, limited overlap with CGRP^+^ or IB4^+^ populations suggests subtype-specific post-transcriptional modulation or restricted downstream signaling. Notably, the preferential production of CBLN2 in large and medium-diameter neurons raises the question of how it exerts functional effects on small-diameter nociceptors. In the central nervous system, CBLN2 is secreted by presynaptic neurons and binds to postsynaptic glutamate receptor delta subunits (GRID1/2) via neurexin (NRXN) intermediaries, competitively modulating neuroligin-mediated pathways to regulate synaptogenesis and plasticity [33, 34]. By analogy, CBLN2 may be locally released within the DRG to act in a paracrine fashion on neighboring nociceptors or in an autocrine manner on neurons of various sizes.

Functionally, siRNA-mediated knockdown of *Sox11* or *Cbln2* significantly attenuated neuropathic pain behaviors, underscoring the therapeutic relevance of this axis. Beyond direct regulation of *Cbln2*, SOX11 may influence broader transcriptional programs through interactions with downstream factors such as ATF3 and c-JUN [13, 16], both of which are well-established contributors to neuropathic pain following nerve injury [37, 38]. Thus, SOX11 may orchestrate complex gene regulatory networks that sustain nociceptive sensitization.

Importantly, SOX11 expression remains elevated through at least 21 days post-SNL, suggesting a sustained role in chronic pain maintenance. This prolonged activity raises the possibility of cooperation with epigenetic regulators such as DNMT3a [39], chromatin remodelers, or non-coding RNAs. Future studies aimed at mapping the SOX11 interactome and epigenomic landscape in injured DRG neurons will be critical to uncovering the full scope of its regulatory influence.

### NF-κB Signaling in CBLN2-induced Nociceptive Sensitization

RNA-seq profiling following CBLN2 administration identified TNF/NF-κB signaling as the most significantly enriched pathway, with 10 chemokines forming an interconnected functional network. This chemokine expression pattern closely mirrors cytokine signatures implicated in the initiation and maintenance of neuropathic pain [40]. The rapid onset of behavioral hypersensitivity (within 1–6 h) suggests that CBLN2 activates NF-κB through non-genomic mechanisms, likely via phosphorylation of IκB kinases [41]. At the molecular level, CBLN2 administration increased p-p65 levels in DRG neurons, an effect reversed by NF-κB inhibitor PDTC. Although PDTC effectively alleviated SNL-induced hypersensitivity, its short half-life (~3 h) highlights the need for more durable NF-κB-targeting therapeutics.

The ability of CBLN2 to trigger NF-κB activation and proinflammatory cytokine upregulation in DRG neurons suggests a trans-neuronal signaling cascade bridging large, medium- and small-diameter sensory neurons. We speculate that CBLN2 may bind to receptors on small-diameter nociceptors, initiating intracellular Ca^2^⁺ signaling or kinase activation that culminates in p65 phosphorylation [41, 42], but we do not exclude the possible role of CBLN2 on large and medium-sized DRG neurons. Initial cytokine release may function in an autocrine or paracrine manner, amplifying NF-κB activation across neighboring neurons. Notably, PDTC not only reversed CBLN2-induced hyperalgesia but also normalized neuronal excitability, underscoring NF-κB’s pivotal role in modulating pain. NF-κB may exert these effects through multiple mechanisms, including the transcriptional upregulation of ion channels such as Nav1.7 [43, 44], Nav1.8 [45], TRPV1 [46, 47], and voltage-gated K channels [48] or through the promotion of proinflammatory cytokine signaling [49, 50].

The chemokine cluster induced by CBLN2, comprising CXCL2, CXCL3, CXCL9, CCL3, and CCL4, likely contributes to a self-sustaining neuroinflammatory loop. In related pain states, CXCL2 derived from Schwann cells promotes macrophage recruitment and nociceptive sensitization via CXCR2 [51], while microglial CXCL3/CXCR2 and CXCL9/CXCR3 interactions amplify inflammatory responses [52, 53]. Similarly, CCL4/CCR5 signaling has been implicated in neuronal hypersensitivity [54], and CCL3 activation of CCR1 on DRG neurons induces Ca^2^⁺ influx and PKC-dependent nociceptive sensitization [55]. These examples illustrate the multicellular complexity of chemokine-receptor networks driving neuropathic pain.

Although our study primarily focused on neuronal mechanisms, the released CBLN2 and the chemokines induced by the CBLN2/NF-κB axis are likely to recruit and activate non-neuronal cell types such as satellite glial cells, macrophages, and T cells [56–58]. These interactions create a self-reinforcing inflammatory microenvironment within the DRG. The suppression of chemokine expression by both PDTC and *Sox11* siRNA highlights the dependence of these inflammatory signals on the SOX11-CBLN2-NF-κB pathway. Given the neuronal specificity of CBLN2 expression, we propose a paracrine model wherein chemokines released by DRG neurons act on adjacent glial and immune cells to perpetuate inflammation. This is consistent with recent single-cell transcriptomic studies demonstrating neuron-to-glia chemokine signaling in chronic pain states [5].

In conclusion, our findings define a novel SOX11-CBLN2-NF-κB signaling cascade that contributes to neuropathic pain through two complementary mechanisms: direct sensitization of sensory neurons and the orchestration of neuroinflammatory responses, indicating that this axis is a promising therapeutic target. Future efforts to identify the CBLN2 receptor and its signaling partners will be crucial to fully understand its mechanism of action.

## Data Availability

All data generated in this study are included in this article. Source data are provided with this paper or upon request from the corresponding authors.
